# Atypical chemokine receptor CCRL2 is overexpressed in prostate cancer cells

**DOI:** 10.7555/JBR.32.20170057

**Published:** 2018-01-05

**Authors:** Niradiz Reyes, Ines Benedetti, Juan Rebollo, Oscar Correa, Jan Geliebter

**Affiliations:** 1. Department of Basic Sciences, School of Medicine, University of Cartagena, Cartagena, Bolívar, Colombia; 2. Research Group of Genetics and Molecular Biology; 3. Research Group of Histopathology; 4. School of Medicine, Department of Microbiology and Immunology, New York Medical College, Valhalla, NY 10595, USA.

**Keywords:** chemokine receptor, prostatic neoplasms, CCRL2 receptor, real-time polymerase chain reaction, tissue array analysis

## Abstract

Atypical chemokine receptors have recently emerged as important molecular players in health and diseases; they affect chemokine availability and function and impact a multitude of pathophysiological events, including the tumorigenesis process. This family of atypical receptors comprises five members: ACKR1/DARC, ACKR2/D6, ACKR3/CXCR7, ACKR4/CCRL1, and ACKR5/CCRL2. This work evaluated the differential expression of these receptors in prostate cancer using quantitative PCR. Further evaluation of CCRL2 at the protein level confirmed its overexpression in a metastatic cell line and in malignant prostatic tissues from patients. CCRL2, a presumed member of the atypical chemokine receptor family, plays a key role in lung dendritic cell trafficking to peripheral lymph nodes. Recent studies have reported the expression of CCRL2 in different human cancer cell lines and tissues. However, its function and expression in prostate cancer has not been previously addressed.

## Introduction

Chemokines are a group of structurally homologous chemotactic molecules that control cell migration and positioning throughout development, homeostasis, and inflammation^[1–4]^. These molecules function by signaling through specific chemokine receptors, a group of ~20 rhodopsin-like seven-transmembrane-spanning receptors in humans and mice^[5–6]^. Chemokine receptors fall into two phylogenetically related subgroups: a larger subgroup of G protein-coupled leukocyte chemotactic receptors and a smaller subgroup of atypical chemokine receptors (ACKRs) that do not signal through G proteins and lack chemotactic activity^[6–10]^. Currently, the ACKR family comprises five receptors: ACKR1 (previously *Duffy Antigen Receptor for Chemokines*, DARC), ACKR2 (formerly D6 or CCBP2), ACKR3 (alias CXCR7), ACKR4 (formerly CCRL1 and CCXCKR), and CC-Chemokine Receptor-like 2 (provisionally designated ACKR5 pending confirmation of their chemokine binding specificity and atypical signaling properties)^[7–8,11]^. ACKRs affect chemokine availability and function, impacting a multitude of pathophysiological events and emerging as important molecular players in health and diseases^[12–15]^. ACKRs may play a role in tumorigenesis, and their effects are determined by the type of cell on which they are expressed and by the co-expression with other chemokine receptors. They are considered as key components of the regulatory network of inflammation and immunity in cancer and may have a major effect on anti-inflammatory and immunotherapeutic strategies^[11,14,16]^.

Chemokine CC-motif receptor-like 2 (CCRL2), a presumed member of the ACKR family and 7-transmembrane G protein-coupled receptor, plays a key role in lung dendritic cell trafficking to peripheral lymph nodes^[17]^. The canonical DRYLAIV motif essential for signaling has been changed to QRYLVFL in this molecule; accordingly, CCRL2 is unable to couple with G-proteins and fails to induce classical chemokine signaling^[11]^. It has been shown that endothelial cells express CCRL2 in a tissue- and activation-dependent fashion, and its induction is regulated by the NF- κB and JAK/STAT signaling pathways^[18]^. Recent studies have reported the expression of CCRL2 in human cancer cell lines and tissues from breast^[19]^, colon^[20]^, glioblastoma^[21]^, and salivary adenoid cystic carcinoma^[22]^. The function and expression of this atypical receptor in prostate cancer has not been previously addressed. Here, we report that CCRL2 expression level is elevated at the mRNA and protein level in prostate cancer cell lines and prostate cancer tissues. The biological role of CCRL2 in prostate cancer warrants further investigations.

## Materials and methods

### Cells

Human bone metastasis-derived prostate cancer PC-3 cells (ATCC® CRL-1435) and non-tumorigenic human prostatic epithelial PWR-1E cells (CRL-11611) were obtained from ATCC (Manassas, VA, USA). PC-3 cells were routinely maintained in phenol red-positive F-12K modified medium (ATCC, USA) containing 10% FBS and 1% penicillin-streptomycin. PWR-1E cells were maintained in keratinocyte serum-free medium (Life Technologies, USA) supplemented with 50 μg/mL bovine pituitary extract, 5% L-glutamine, and 5 ng/mL epidermal growth factor. Cells were grown as monolayers in T-25 tissue culture flasks, in a humidified atmosphere containing 5% CO_2_ at 37°C and passaged once/twice a week. For all the experiments, cells were harvested at low passage numbers: PC-3 cells between passages 28 and 31, and PWR-1E between passages 18 and 22.

### RNA extraction and cDNA synthesis

Total RNA was isolated from PC-3 and PWR-1E cells grown to approximately 80% confluence with TRI-Reagent (Ambion, Austin, TX, USA) following the manufacturer’s instructions. Concentration and purity of total RNA was assessed by spectrophotometry at 260/280 ratio with NanoDropTM (Thermo Scientific, USA). RNA samples from cell lines were processed for reverse transcription using the QuantiTect Reverse Transcriptase kit (Qiagen, Germantown, MD, USA). First, to eliminate any contaminating genomic DNA, 1 μg of total RNA was incubated with genomic DNA Wipeout Buffer (Qiagen, USA) for 2 minutes at 42°C. The reverse-transcription master mix, containing QuantiTect RT enzyme, QuantiTect RT Buffer and RT Primer Mix (oligo-dT and random primers), was prepared and added to the template RNA. Samples were incubated at 42°C for 15 minutes, followed by inactivation at 95°C for 3 minutes. A 20 μL final volume of cDNA was stored at −20°C until used in qPCR.

### Quantitative PCR 

Transcript expression profiles for the currently described atypical chemokine receptor family were analyzed in the cell lines by quantitative PCR (qPCR) using QuantiTect SYBR Green PCR Master Mix (Qiagen, USA) and qPCR primer panels (OriGene Technologies, USA). Primers included in the qPCR primer panels are shown in ***Table 1***. PCR for each sample was performed in triplicates with QuantiTect® SYBR® Green PCR Master Mix (Qiagen, USA) in a StepOne Real-Time PCR System (Applied Biosystems, USA), with an initial denaturing step at 95°C for 15 minutes, followed by 40 cycles of amplification. Changes in gene expression for each target gene in metastatic PC-3 cell line were calculated with the Sequence Detection System 2.1 software (Applied Biosystems, USA) using the comparative CT method (2^–^^ΔΔ^^CT^)^[23]^ and the reference non-tumorigenic cell line PWR-1E. Expression levels for each target gene were normalized to the expression levels of the reference genes *HPRT1 *and ** β *-actin*. Melting curves for all samples were acquired for quality control purposes. Statistical significance of differences in gene expression was calculated with the *t*-test using GraphPad Prism (GraphPad Software Inc, San Diego, CA, USA). A *P*<0.05 was considered statistically significant.

**Tab.1:** Primer sequences for PCR assays

Atypical chemokine receptors	Primer pairs (Forward/Reverse)
ACKR1 (DARC)	5'-GGGCTGAAGAAGGCATTGGGTA /CTTGGACCTCACCAGGAAATCC-3'
ACKR2 (D6)	5'-GACTACGCACTCCAGGTAACAG / AAGCCTTCAGGTACTGGCGGAA-3'
ACKR3 (CXCR7)	5'-CCAAGACCACAGGCTATGACAC / TGGTTGTGCTGCACGAGACTGA-3'
ACKR4 (CCRL1)	5'-GTCTCTGGAATGCAGTTTCTGGC / GGTATGCTCAGCAAGATGGCAG-3'
ACKR5 (CCRL2)	5'-TGCCGCTGTTTCCATCTGCGTA / ACACTTCGGTGGAATGGTCAGG-3'

### Immunocytochemistry

Detection of CCRL2 was performed with anti-human-CCRL2 antibody (ab167114, abcam®) and EXPOSE Mouse and Rabbit Specific HRP/AEC Detection IHC system (ab93686, abcam®). Briefly, cells (1×10^4^ cells/mL) were seeded on glass bottom Petri dishes (FluoroDish TM, World Precision Instruments, Inc, USA) in culture medium overnight. On reaching ~70% confluency, the dishes were rinsed twice with PBS and fixed in 4% paraformaldehyde/PBS for 10 minutes, and washed three times in PBS. Unspecific binding sites were blocked with protein block reagent for 10 minutes at room temperature. Primary mouse/IgG, monoclonal, anti-human-CCRL2 antibody diluted in UltraClean Diluent (Thermo Scientific, USA) (1:100) was added and incubated at 4°C overnight. Then, dishes were washed for 5 minutes three times with PBS-T, incubated 10 minutes with Mouse Specifying Reagent, rinsed 2 times in buffer, and HRP conjugate applied and incubated for 15 minutes at room temperature. After washing, antibody binding was detected with AEC chromogenic substrate (3-amino-9-ethyl-carbazole). Negative control slides were incubated in absence of primary antibody only. Nuclei were counterstained with hematoxylin (Sigma-Aldrich), dehydrated, and mounted. Intensity of staining was evaluated at 100× and 400× final magnification with an Eclipse 400 microscope connected to a DSFi1 camera (Nikon, Japan) by a pathologist who was blinded to the cell type on each slide. Staining intensity was scored using NIS-Elements-3.0® software and classified as strong, moderate, weak, or absent.

### Selection of specimens for inclusion into the TMA and ethical considerations

CCRL2 protein expression was evaluated using a prostate tissue microarray (TMA) approach. For TMA construction, archived FFPE radical prostatectomy and trans-urethral prostatectomy tissue specimens were obtained from 47 patients with a previous diagnosis of localized prostate cancer, who underwent surgical resection as their primary treatment. For each patient, hematoxylin- and eosin-stained sections from donor blocks were subjected to pathological review to determine the presence of benign and cancerous tissue; then, matched tissue sections for benign prostate tissue (BPT) and cancerous prostate tissue (PCa) were cut out from the respective FFPE block for TMA. For each case, matched BPT sections were taken from regions distant to the PCa lesions. FFPE tissue specimens were obtained from the archives of the Department of Pathology at Hospital Universitario del Caribe with approval by local ethics committee and the studies were conducted according to the Declaration of Helsinki.

### TMA design and construction

TMA design and construction were performed at the Sidney Kimmel Cancer Comprehensive Center, Johns Hopkins University (Baltimore, MD, USA). TMA construction was performed according to a previously described technique, using 0.6 mm cores of PCa and matched BPT from donor blocks^[24]^. Four μm sections were cut off from the TMA block and stained with H&E to check the histopathology in each tissue core according to the previous design in the TMAJ^®^ software. TMA processing was performed at Johns Hopkins University Oncology Tissue Services Core, Regional Oncology Research Center (Baltimore, MD, USA). The number of cores grouped by tissue type was 95 BPT and 136 PCa for a total of 231 cores.

### TMA immunohistochemistry and data analysis

To evaluate CCRL2 protein expression in PCa and BPT, 4 μm sections from the TMA block were subjected to immunohistochemistry. TMA sections were deparaffinized in xylene and rehydrated in alcohol according to standard procedures. Sections were steamed for 30 minutes in 10 mmol/L citrate buffer (pH 6.0) to unmask the epitopes, incubated in Hydrogen Peroxide Block and Protein Block (Abcam^®^, Cambridge, MA, USA), and then incubated with primary mouse/IgG, monoclonal, anti-human-CCRL2 antibody (ab167114, abcam®) diluted in UltraClean Diluent (Thermo Scientific, USA) (1:100) at 4°C overnight. Then, slides were washed for 5 minutes three times with PBS-T, incubated 10 minutes with Mouse Specifying Reagent, rinsed 2 times in buffer, and HRP conjugate applied and incubated for 15 minutes at room temperature. After washing, antibody binding was detected with AEC chromogenic substrate (3-amino-9-ethyl-carbazole). Negative control slides were incubated in absence of primary antibody. All tissue slides were counterstained with hematoxylin, dehydrated, and mounted. Images from stained samples were captured at 100× and 400× final magnification using an Eclipse 400 microscope connected to a DS-Fi1 camera (Nikon, Japan). To quantify CCRL2 expression in the TMA, immunostaining was scored by microscopically assessing the percentage of luminal epithelial cells with positive staining to obtain a final H-Score. For this, 10 fields were chosen at random at 100× magnification and staining intensity in the benign luminal epithelial cells and tumor cells was scored as 0, 1, 2, or 3, corresponding to negative-, weak-, intermediate-, and strong-brown staining, respectively. The total number of cells in each field and the number of cells stained at each intensity score were counted. The average percentage positive was calculated and the following formula was applied: H-score= (% of cells stained at intensity category 1×1) + (% of cells stained at intensity category 2×2) + (% of cells stained at intensity category 3×3).

### Statistical analysis

Differences in CCRL2 protein expression were assessed by comparing IHC scores (H-Scores) between BPT and PCa groups using Mann-Whitney test for non-Gaussian distribution. Statistical analyses were performed using GraphPad Prism^®^ v5.00 software (GraphPad Software Inc, San Diego, CA); *P*< *0.05* was considered statistically significant. Immunostaining intensity scoring in BPT and PCa regions was performed by two independent pathologists in blind.

## Results

### mRNA expression of atypical chemokine receptors in PC-3 cells

Expression data for the atypical chemokine receptors analyzed by qPCR in PC-3 cells are presented in ***Table 2***. Compared with PWR-1E cells, two out of five atypical chemokine receptor genes were overexpressed (ACKR3/CXCR7 and CCRL2) and the remaining three were underexpressed (ACKR1/DARC, ACKR2/D6, and ACKR4/CCRL1) in PC-3 cells.

**Tab.2:** Expression data analyzed by quantitative PCR in PC-3 cells

Atypical chemokine receptors	PWR-1E CT	PC-3 CT	FC	*P*-value
ACKR1(DARC)	31.95	ND	NA	
ACKR2(D6)	33.95	34.95	-4.70	0.0002
ACKR3 (CXCR7)	33.95	29.93	6.86	0.003
ACKR4(CCRL1)	30.95	32.94	-9.34	0.001
ACKR5 (CCRL2)	36.99	24.95	6758.56	0.0001

Note: Fold-change (FC) values were calculated using the 2 ^−ΔΔCT^ method as described in Materials and methods. For fold changes<1, the negative inverse of the result was reported as a fold decrease. *P* values were calculated by Student’s *t*-test (two sided, for triplicate samples) from comparing fold changes in PC-3 cell lines relative to PWR-1E cell line. Expression levels for each target gene were normalized to the expression levels of the reference (housekeeping) genes *HPRT1* and β *-actin*. Gene transcripts with CT (threshold cycles)>37 were considered not detected (ND).

### Immunocytochemistry of CCRL2 in cell lines

**Fig.1:**
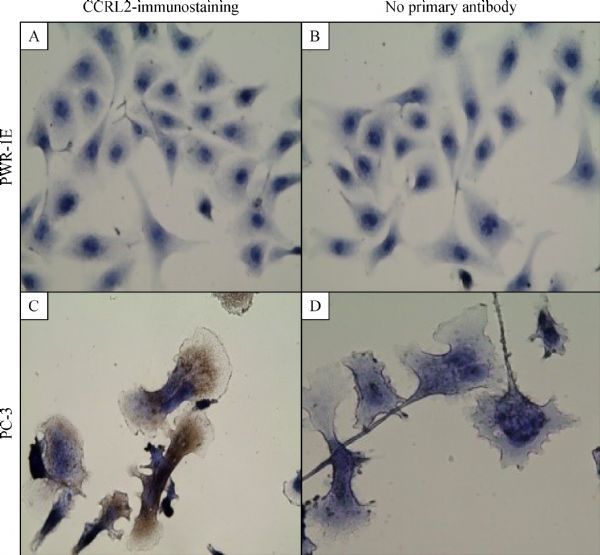
**Immunocytochemical staining reveals overexpression of CCRL2 in PC3 cells.** Cells were incubated with mouse/IgG, monoclonal,anti-human-CCRL2 antibody followed by the respective secondary antibody. The detection was performed with EXPOSE Mouse and Rabbit Specific HRP/AEC Detection IHC system. PWR-1E cells with negative cytoplasmic staining (A). PC3 cells with strong cytoplasmic staining (C). Cells stained with no primary antibody served as negative controls for each cell line: PWR-1E (B); PC3 (D); Magnification: 400×; Brown color: AEC; Blue color: hematoxylin counterstain.

As mRNA for CCRL2 exhibited the most differential expression, it was chosen for further analysis at the protein level in the prostate cancer cell line PC-3 and the non-tumorigenic PWR1-E cells. Immunocytochemical staining revealed overexpression of CCRL2 in PC3 cells compared with the non-tumorigenic PWR-1E cells ( ***Fig. 1***). While non-tumorigenic PWR-1E cells did not exhibit cytoplasmic staining, PC3 cells had a strong cytoplasmic staining, indicating a high level of expression. The data indicated that CCRL2 staining was significantly stronger in the cancerous prostate cell line, compared to the non-tumorigenic cell line.

### CCRL2 protein expression in human prostate tissue microarray

**Fig.2:**
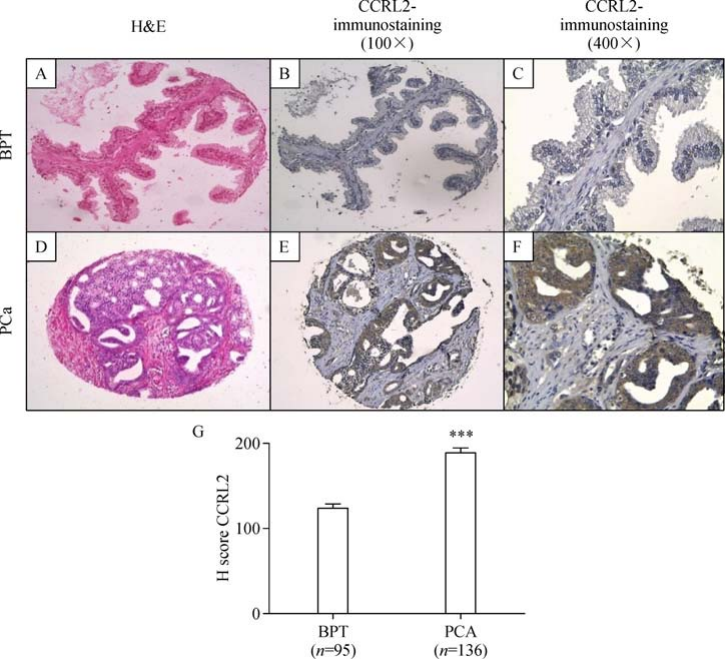
**Immunohistochemical staining of CCRL2 in BPT and PCa in a human prostate tissue microarray. ** BPT in a prostatectomy tissue spot with H&E staining 100×(A), immunohistochemical staining of CCRL2 showing absent to mild staining of CCRL2 in the epithelial compartment (B and C). Prostate cancer (PCa) tissue in a prostatectomy tissue spot with H&E staining 100×(D), immunohistochemical staining of CCRL2 showing strong staining in tumor compartment (E and F). H-score of immunohistochemical (IHC) staining in prostate tissues (G). Hscores were calculated as described in Materials and methods. *** *P* < 0.0001, Mann Whitney test.

CCRL2 protein expression was evaluated by IHC in a TMA arrayed with 231cores from benign prostate tissue and prostate cancer tissue obtained from 47 patients. Immunohistochemical evaluation showed that CCRL2 staining was present predominantly in the cytoplasm of prostate cancer tumor cells. Compared with benign epithelial cells in BPT, CCRL2 expression was higher in epithelial tumor cells in cores with PCa ( *P*<0.0001). Mann–Whitney test showed a significant difference in staining between benign and cancerous tissues ( *P*<0.0001). There was a higher mean in immunohistochemical staining score in PCa (189.0, 95% CI: 176.6-201.4), compared with BPT (124.3, 95% CI: 115.0-133.6). The results of CCRL2 immunostaining in TMA are presented in ***Fig. 2***.

## Discussion

The aim of this study was to investigate the expression pattern of atypical chemokine receptors in prostate cancer cell lines and tissues. Our qPCR results showed differential expression of ACKRs in PC-3 metastatic cells compared with non-tumorigenic PWR-1E cells. ACKR1 (DARC) was undetected, while ACKR2 (D6) and ACKR4 (CCRL1) were underexpressed in PC-3 cells. DARC functions by binding CXC chemokines that possess an *N*-terminal sequence (Glu-Leu-Arg), referred to as the ELR motif^[25]^. Several studies have reported that the ELR motif is responsible for the angiogenic properties of CXC chemok-ines^[26–27]^. Expression of CXC chemokines bearing the ELR motif has been shown in prostate cancer cells, and elevated levels have been found in tumors from prostate cancer patients^[28]^. Thus, lack of DARC expression in aggressive PC-3 cells may favor growth or metastasis due to the activity of angiogenic chemokines, as has been suggested for breast cancer^[29]^. Similarly, regarding the expression of D6, the experimental results suggest that D6 is a negative regulator of growth in lung cancer, mainly by the sequestration of specific chemokines^[30]^. In contrast, the function of ACKR4 (CCRL1) *in vivo* is still unclear. This atypical chemokine receptor is able to bind and sequester homeostatic chemokines such as CCL19, CCL21, and CCL25, without exhibiting any classical chemokine signaling. Since these chemokines are involved in cancer development and metastasis, ACKR4 might be able to inhibit cancer cell proliferation and invasion^[11,31]^.

On the other hand, PC-3 cells in this study showed overexpression of atypical chemokine receptors ACKR3 (CXCR7) and ACKR5 (CCRL2). CXCR7 is a dual specificity receptor that binds with high affinity to CXCL12 and CXCL11 chemokines^[11]^; with its upregulation reported in different tumor types, CXCR7 may play a role in tumor cell growth, survival, and metastasis^[32–33]^. It is also expressed at high levels on tumor-associated vasculature, suggesting its important role in tumor angiogenesis^[34]^. Expression of CCRL2 has been previously reported in cell lines and tissues from breast^[19]^, glioblastoma^[21]^, salivary^[22]^, and colorectal origin^[20]^. The function and expression of CCRL2 in cancer are not understood at present. Depending on the tissue type, it may suppress tumor^[19]^ or promote cell migration and invasion^[21]^. Currently, data on the expression of CCRL2 and its involvement in prostate cancer is lacking. Our results show that metastatic prostate cancer cell line PC-3 significantly expresses higher mRNA and protein levels of CCRL2 compared with non-tumorigenic PWR-1E. CCRL2 showed cytoplasmic staining in PC-3 cells, in agreement with data in the Human Protein Atlas^[35]^, regarding the cytoplasmic expression of this protein with a granular pattern in most tissues.

In addition, immunohistochemistry of prostate cancer tissue specimens in TMA revealed that CCRL2 expression was significantly stronger in epithelial cells of cancerous acini than in those of matched adjacent benign acini from the same patient. To this point, the physiologic effects of CCRL2 expression in epithelial cancer cells are unclear.

Here, we report for the first time an elevated expression of chemokine receptor CCRL2 in prostate cancer cell lines and tissue samples. The key findings of this study constitute a starting point for subsequent research on the biological role and clinical implications of CCRL2 expression in prostate cancer. A clear understanding of the downstream signaling pathway requires additional studies, and the potential applications of CCRL2 as a therapeutic target or biomarker in PCa deserve further research.
